# Case of Insanity Cured by a Surgical Operation

**Published:** 1845-09

**Authors:** 


*Case of Insanity cured by a Surgical operation.*—A gentleman engaged in the higher departments of trade—a good man, and an affectionate parent—had two sons, who at the time I begin their history, were respectively of the ages of five and ten. The attachment between them was so remarkable as to be the common topic of conversation among their friends and acquaintance. The children were always together; and to see them walk round the garden, with the arm of the elder round the neck of the younger, while the other who could not reach his neck, endeavored to clasp his waist—with their long auburn hair, in the fashion of the day, hanging down in ringlets, and as the elder stooped to kiss his little brother covering his face, those who had seen them thus occupied, their lovely features beaming with affection, would have said, that nothing on earth could give a more vivid idea of angels.

The children when separated for a few hours were miserable; and when the time arrived for sending the elder to school, it was a subject of serious reflection with the parents and friends, whether so intense an affection should be checked or encouraged: the former was decided on, and the elder was sent to a distance.

Both children were so exceedingly unhappy, that sleepless nights, loss of appetite, incessant weeping, and rapid wasting of body, made every one fearful of the consequences of prolonging the absence, and they were brought together again. Those who witnessed the tumultuous joy of their meeting, describe it as inexpressibly affecting. They soon recovered their health and spirits, and their mutual affection seemed if possible to be increased by their temporary separation.

The experiment, after a while, was again made, with similar results; and it was decided never to risk another.

An arrangement was now entered into with a school-master to receive both boys, although contrary to the regulations of his establishment, which psofessed to admit none under ten years of age.

The two boys kept themselves almost entirely aloof from all the rest; the elder helped the younger in his education, watched him with a kind of parental solicitude, kept a vigilant eye upon the character of boys who sought his society, and admitted none to intimacy with his brother of whom he did not entirely approve. The slightest hint of his wish sufficed with the younger, who would almost as soon have contemplated deliberately breaking the Commandments, as opposing his wishes in the slightest degree.

Both made rapid progress in their education, and their parents' hearts were filled with thankfulness for the blessing.

In the midst of this happiness, news arrived from the schoolmaster that, from some unexplained cause, the elder boy had begun to exercise a very unreasonable and tyrannical authority over the younger; that he had been repeatedly punished for it; but although he always promised amendment, and could assign no cause, reasonable or unreasonable, for his conduct he soon relapsed into his usual habits, and the schoolmaster requested to know what was to be donc. The father immediately sent for both boys, and entered upon a lengthy investigation. The little one was almost heart-broken, and exclaimed, "He might beat me every day if he would but love me; but he hates me, and I shall never be happy again."

The elder could assign no reason for his animosity and ill-treatment; and the father, after many remonstronces, thought it right to inflict on him very severe corporeal chastisement, and confined him to his room for some days with nothing but bread and water. The lad on his liberation gave solemn promises of altered conduct, but shewed little affection for his brother, although the latter used a thousand innocent stratagems to inspire him with tenderness. They returned to school. In a few days similar scenes and worse occurred; the boy was again and again punished by the master, again and again promised amendment, but in vain, and he was at last taken away from school by his father.

A repetition of severe punishment, long incarceration, and a rejeetion by all his relatives, had no effect in changing his disposition; his dislike to his brother became fixed animosity, and from animosity degenerated into the most deadly hatred: he made an attempt on the child's life; and if he saw him pass an open door, would throw a carving-knife at him with all the fury of a maniac.

The family now resorted to medical advice, and years passed in hopeless endeavors to remove a disposition obviously depending on a diseased brain. Had they taken this step earlier, these floggings and imprisonments would have been spared, as well as the heart-sickening remorse of the father.

Still the boy was not insane: on every topic but one he was reasonable, but torpid; it was only by the sight of his brother, or the sound of his name, that he was aroused to madness. The youth now advanced towards manhood. When about the age of fifteen he was taken with a violent but Platonic passion for a lady more than forty years of age, and the mother of five children, the eldest older than himself. His paroxysms of fury now became frightful; he made several attempts to destroy himself; but in the very torrent and whirlwind of his rage, if this lady would allow him to sit at her feet and lay his head on her knee, he would burst into tears and go off into a sound sleep, wake up perfectly calm and composed, and looking up into her face with lack-lustre eye, would say, "Pity me; I can't help it."

Soon after this period he began to squint, and was rapidly passing into hopeless idiocy, when it was proposed by Mr. Cline to apply the trephine, and take away a piece of bone from the skull, in a place where there appeared to be a slight depression. "The indication is very vague," said he, "and we should not be justified in performing the operation but in a case in which we cennot do any harm; he must otherwise soon fall a sacrifice."

It was done, and from the under surface grew a long spicula of bone piercing the brain! He recovered, resumed his attachment to his brother, and became indifferent to the lady.

The disease which led to these terrible results had its origin in a blow on the head with the end of a round ruler—one of the gentle reprimands then so common with schoolmasters.—*Journal of Insanity.*

